# Supplementary material for: Audible Feedback Improves Internal Model Strength and Performance of Myoelectric Prosthesis Control
Source: Sci Rep. 2018 Jun 4;8:8541. doi: 10.1038/s41598-018-26810-w (PMC5986794; doi:10.1038/s41598-018-26810-w)
Supplement: Supplementary file 1 — Appendix [file 41598_2018_26810_MOESM1_ESM.pdf]

Audible Feedback Improves Internal Model Strength and Performance of Myoelectric Prosthesis Control

Ahmed W. Shehata[1,2,*], Erik J. Scheme[1,2], and Jonathon W. Sensinger[1,2]

[1]University of New Brunswick, Electrical and Computer Engineering, Fredericton, E3B5A3, Canada
[2]Institute of Biomedical Engineering, Fredericton, E3B5A3, Canada
[*]ahmed.shehata@unb.ca

Supplementary Material

Appendix

Outcome measure	Controller learning effect	Dependent paired t-test
Adaptation rate	FLT effect on AUG	t (3) = -2.1, p = 0.17
	AUG effect on RAW	t (3) = -1.7, p = 0.34
	AUG effect on FLT	t (3) = -0.20, p = 0.86
	RAW effect on AUG	t (3) = -0.61, p = 0.65
JND	FLT effect on AUG	t (3) = 0.50, p = 0.66
	AUG effect on RAW	t (3) = 3.0, p = 0.090
	AUG effect on FLT	t (3) = 5.7, p = 0.029
	RAW effect on AUG	t (3) = -1.6, p = 0.26
Internal model uncertainty	FLT effect on AUG	t (3) = 1.9, p = 0.20
	AUG effect on RAW	t (3) = -0.0070, p = 1
	AUG effect on FLT	t (3) = 1.4, p = 0.25
	RAW effect on AUG	t (3) = 0.38, p = 0.73
On-axis target path efficiency	FLT effect on AUG	t (3) = 2.2, p = 0.30
	AUG effect on RAW	t (3) = -3.0, p = 0.10
	AUG effect on FLT	t (3) = -1.45, p = 0.38
	RAW effect on AUG	t (3) = -14, p = 0.005
On-axis target accuracy	FLT effect on AUG	t (3) = 0.03, p = 0.98
	AUG effect on RAW	t (3) = -1.7, p = 0.23
	AUG effect on FLT	t (3) = 23, p = 0.028
	RAW effect on AUG	t (3) = -0.96, p = 0.44
Off-axis target path efficiency	FLT effect on AUG	t (3) = 0.61, p = 0.61
	AUG effect on RAW	t (3) = -1.7, p = 0.24
	AUG effect on FLT	t (3) = 0.74, p = 0.54
	RAW effect on AUG	t (3) = 0.61, p = 0.61
Off-axis target accuracy	FLT effect on AUG	t (3) = -1.5, p = 0.27
	AUG effect on RAW	t (3) = 0.95, p = 0.44
	AUG effect on FLT	t (3) = 2.5, p = 0.13
	RAW effect on AUG	t (3) = -2.8, p = 0.11

Table A1. Statistical analysis using dependent t-test for subgroups 2, 4, 5, and 6 to investigate possible learning effect.

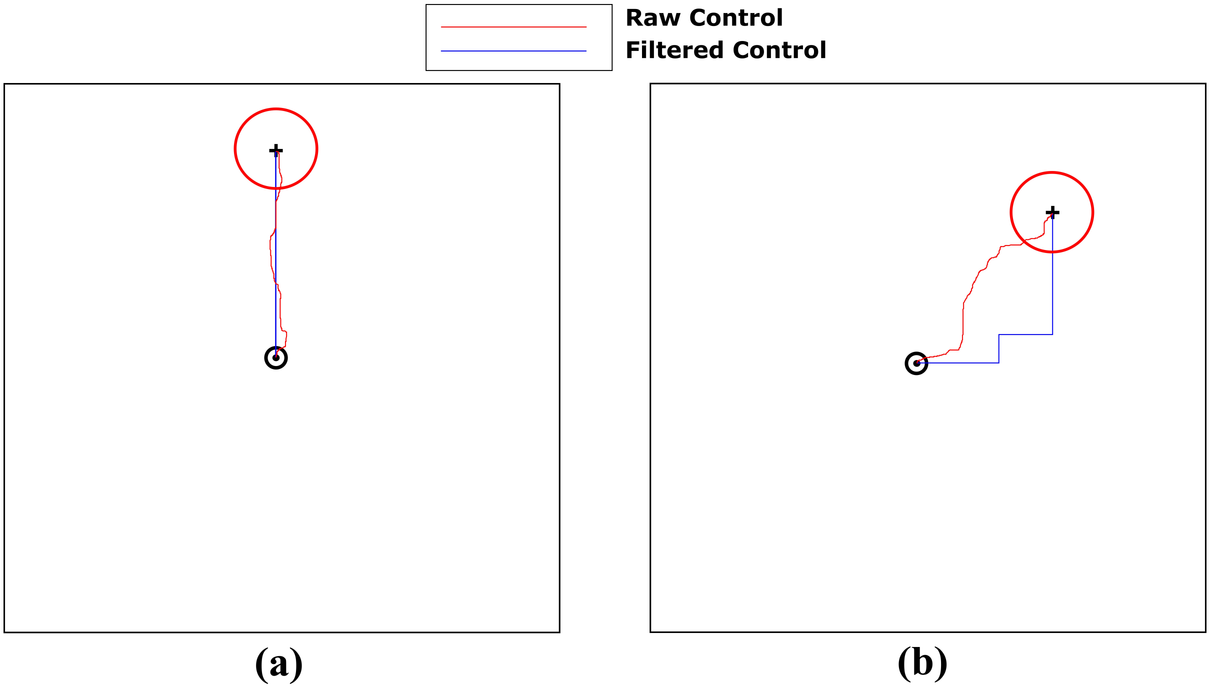

Figure A1. Sample trajectory of using Raw control and Filtered control to reach (a) on-axis target (b) off-axis target. Raw Control trajectory in red and filtered control trajectory in blue.
